# The Scottish Women’s Health Fair, 1983: A Showcase of the Scottish Women’s Health Movement^1^

**DOI:** 10.1353/hah.2024.a952498

**Published:** 2024

**Authors:** Karissa Robyn Patton

**Affiliations:** University of Edinburgh

**Keywords:** women’s health, feminist praxis, health activism, Scottish feminism, health politics

## Abstract

In May 1983 women from across Scotland came together in Edinburgh for the Scottish Women’s Health Fair (SWHF). Organised by and for ‘ordinary’ women, the SWHF offered a substantive two-and-half day programme—including information sessions, film screenings, workshops, and information stall—that attracted over two thousand attendees. A diverse group of organisers, presenters, and attendees at the SWHF applied concepts of holistic and social health as they discussed a wide range of topics, from women suffering from depression to damp housing and cervical smears. The women who organised the SWHF reflected on this event as a pivotal moment for them personally, and for the women’s health movement in Scotland more broadly. The success of the SWHF was achieved in the face of political and professional controversies that placed Scottish women’s health activists in opposition with the Scottish Home and Health Department and the World Health Organisation European Region. Within the context of these controversies, this article situates the SWHF as a strategic showcase of the strength of the women’s health movement in Scotland and the wider public support of women’s health praxis and ideas.

## Introduction

In May 1983, on a particularly rainy weekend, women from across Scotland came together in Edinburgh for the Scottish Women’s Health Fair. This event (hereafter SWHF, or fair) hosted a packed programme of information sessions, film screenings, and workshops organised into themes of mental health, well women services, and health activist strategies alongside information stalls from groups across the country.^2^ Healthcare professionals, community health researchers, feminist activists, and so-called ‘ordinary women’ from across Scotland joined the fair as presenters and attendees.^3^ The SWHF demonstrates both a local and national example of broader feminist health praxis in the late 1970s and early 1980s. For example, SWHF organisers adopted a community-led approach in the fair’s design as they travelled around Scotland to gather information about what ‘ordinary’ Scottish women wanted on the agenda. The diverse group of organisers, presenters, and attendees at the SWHF applied concepts of holistic and social health as they discussed a wide range of topics, from women suffering from depression to damp housing and cervical smears.^4^ The women who organised the SWHF reflected on this event as a pivotal moment for them personally and for the women’s health movement in Scotland more broadly.^5^

The fair’s significance, however, is more than the organisers’ utility of feminist health philosophies and approaches. Framed within the local, national, and international controversies and deliberations about women’s health, SWHF organisers strategically distinguished themselves from government and international health organisations and positioned the fair as representative of ‘ordinary’ Scottish women’s health needs. In the early 1980s, the feminist health networks in Edinburgh and Scotland were strategising a response to the censorship of a Scottish Health Education Group (SHEG) publication on women’s health, a book titled *Well Woman: A Guide to Women’s Health*, by the Scottish Home and Health Department. *Well Woman* was censored due to its in-depth discussion of women’s bodies and sexuality and, in particular, the inclusion of an anatomically correct illustration of a vulva, clitoris, and vagina. Despite this censorship, SHEG’s public health materials and programming on women’s health had caught the attention of women’s health educators abroad. In 1982, the World Health Organisation (hereafter WHO) European Region approached SHEG, a civil servant-run public health education department of the National Health Service (hereafter NHS),^6^ about co-hosting a conference on women and health in Scotland. While the ongoing women’s health work in Scotland may have attracted the WHO’s attention, the recent *Well Woman* controversy meant the WHO was entering a precarious political space within Scotland’s women’s health networks. And when the WHO conference, sponsored by SHEG, was announced as an event for invited international delegates only, many others in the women’s health community felt slighted. In the face of these controversies and tensions at the local, national, and international levels, I argue that the SWHF should be understood as a strategic showcase of the strength of the women’s health movement in Scotland and the wider public support of women’s health praxis and ideas.

This article examines the SWHF as both a local and national Scottish event and builds on the scholarship of other historians who use place as a critical lens. Several historians of reproductive health and feminism have advocated the use of local and regional histories to demonstrate how on-the-ground and community-oriented activisms connected and diverged from national movements and policy.^7^ Scholars like Cathy Moran Hajo and Nancy Janovicek have shown that local women’s activism did not always align with the goals of national feminist associations.^8^ Lianne McTavish has argued that when it comes to reproductive healthcare, in particular, examining the disconnects between national legislation and regional service provision illuminates various locally and regionally specific reproductive health inequities.^9^ Hannah Elizabeth’s work ‘demonstrates the gains to be found in paying attention to regionally specific queer activism’ in their investigation of the ‘muddy divide’ between patients, medical professionals, and NHS bureaucrats within Birmingham’s LesBeWell’s queer health publication, *Dykenosis*.^10^ Recent scholarship about national legislations, like Britain’s *Abortion Act*, have demonstrated how various contexts of place—between England and Wales, Scotland and Northern Ireland—affected the development and implementation of laws, and the reactions, across local, regional, and national borders.^11^ Historians of feminism in Scotland, Kristin Hay and Sarah Browne, have argued that a local and regional view of the history of feminism offers more insight into grassroots endeavours across ‘a wide range of women and the ways in which identities and ideas differed depending upon location’.^12^

Using the archival files from the SWHF and oral history interviews with SWHF organisers, I explore the feminist methods and strategies of the SWHF organisers. An examination of oral history interviews with SWHF organisers alongside the meeting minutes, reports, and official programme files from their planning committee demonstrate a Scottish feminist approach to the fair that broadly defined health and wellbeing, and underscored the involvement of ‘ordinary women’ from a wide range of communities across Scotland. In doing so, the SWHF organisers emphasised the dissonance between their event and the invitation-only WHO conference. The SWHF organisers positioned themselves and the fair in response, and even in juxtaposition, to the WHO conference, but their approach and messaging of the fair demonstrate a deep and broad political utility beyond the WHO conference. The framing of the SWHF as by and for ‘ordinary’ Scottish women implicitly but shrewdly demonstrated that the women’s health movement was well in touch with the health wants and needs of Scottish women. While the Scottish Home and Health Department had claimed that the *Well Woman* book was too ‘strident’ for Scottish women, the flood of thousands of ‘ordinary’ Scottish women to the fair underscored broad support for the women’s health information. Building on the local, national, and international contexts of women’s health activism and controversies, I situate the SWHF as a useful case study in the broad political utility of community-led women’s health initiatives in demonstrating support during a time of political and professional opposition.

### Shifting Ground: Medicine, Public Health, and Women’s Health, 1960s−1990s

The late 1960s to the 1990s was an era of policy change, reorganisation, and shifting power dynamics within the UK’s NHS and medicine more broadly. Historian Virginia Berridge’s body of work has shown how society and medicine adapted to concerns about technology, modernity, and power following the Second World War, explaining that the 1970s and 1980s, in particular, saw a rise in new health professions, critiques of medicine, and community care endeavours outside the biomedical model. Epidemiological research from the 1960s and new governmental reports like the Canadian *Lalonde Report* (1974) and the British *Prevention and Health* report (1976) emphasised that individual behaviour was linked to ill-health, resulting in health education and preventative medicine being prioritised in population health strategies. In response to this focus on health education, Berridge explains, official health education offices, including the Health Education Council (England and Wales) and SHEG, emerged.^13^ At the same time, scholars like Michel Foucault and Thomas McKeown questioned the actual impact of biomedicine and science on public health, and activist groups advocated for a more balanced doctor-patient relationship.^14^ Paired with the shifting approach to public health policy and practice, these intellectual and activist critiques of biomedicine trickled down to on-the-ground changes in British health spaces in the 1970s and 1980s.

These broader changes in attitudes towards medicine and health in Britain were linked to women’s health policy, labour, and activism. Historians have pointed to the shifts in health policy in the late 1960s as sparking new debates about medical authority and women’s reproductive autonomy. In 1968, the *Abortion Act* loosened abortion regulation to permit doctors to perform the procedure for physical and mental health reasons, and new policy on contraception ‘enabled local health authorities to provide services on a general basis’.^15^ However, the British Family Planning Service continued to provide birth control services on behalf of the local health authorities until 1975 when contraceptive services were transferred to general practitioners.^16^ These legal and healthcare shifts concerning reproductive health services intersected with the growing women’s liberation movement. Feminists critiqued biomedicine as part of a broader patriarchal society and formed self-help groups to address the gaps they saw in the biomedical model of healthcare. These new feminist health spaces ‘had consequences for the role and function of health care occupations’,^17^ as they offered alternative means to get health information and coached women to advocate for themselves in healthcare spaces.

The SWHF occurred within this context of the broader women’s health movement, the ‘revival’ of official public health policy, and the shifting relationship between medicine and society in the early 1980s. These feminist critiques of biomedicine and the creation of self-help groups were not unique to Britain, but part of the broader international women’s health movement in the Western world. In the United States and Canada, for example, the women’s health movement embraced self-help health education and care as a key part of their activism. As historian Judith Houck has shown, this feminist self-help and health information activism ‘was also an institution-building movement that sought to transform women’s relationship with medicine’.^18^ To achieve this goal, activists created spaces distinct from biomedical healthcare clinics and hospitals, where women could learn about their bodies and increase their autonomy.^19^ In Britain, feminist consciousness raising groups and self-help publications similarly provided a space for women to share their lived experience and their critiques of biomedicine. Sara Crook’s examination of the mental health advocacy of one local north London women’s group, for instance, illustrates how the on-the-ground work of activist groups fuelled ‘grassroots intervention in to women’s mental healthcare’.^20^ In Scotland, women’s health activists ‘argued that doctors had too much power because women knew so little about how their bodies worked’, and created grassroots self-help groups to facilitate women’s bodily autonomy within and outside the medical sphere.^21^

### Situating the SWHF: Community & Controversy surrounding Women’s Health in 1980s Edinburgh and Scotland

By the early 1980s, Scottish feminist activism operated on multiple levels across British, Scottish, and local networks. Many Scottish women were part of broader British feminist efforts to establish Well Women clinics across the UK and lobby for better abortion laws.^22^ Others established specific Scottish campaigns to parallel these broader UK groups.^23^ Hay’s work on the Scottish Abortion Campaign, for example, highlights a moment at the turn of the decade when Scottish women splintered from a broader UK organisation to prioritise their specific Scottish issues. As Hay shows, when women from the National Abortion Campaign called to expand their mandate beyond abortion, Scottish members worried that decentralising abortion in the organisation would hinder their progress in Scotland, where they faced ‘distinctive barriers’ to the procedure. These Scottish women formed the Scottish Abortion Campaign (SAC) in response. Beyond national organisations like the SAC, oral history narrators recall the many women’s health centres and projects in 1980s Edinburgh that focused on localised community needs alongside national networks of women’s health and reproductive rights activists. Women’s community health projects emerged in Edinburgh neighbourhoods, especially working-class areas like Gorgy-Dalry, Pilton, and Craigentinny, and created community-directed women’s health education and support services.^24^ Oral history interviewee Gica Loening described the 1980s as the ‘heyday’ of community projects, and recalled that ‘on International Women’s Day, almost every locality had some kind of women’s event happening.’^25^ While many worked in UK-wide women’s health networks and activities, Scottish feminists established specific community-focused, service-based activism in the 1970s and 1980s to meet their distinct needs.

Beyond abortion campaigns and service-based community health projects, Edinburgh was home to lively feminist groups who offered critiques about the relationship between biomedicine and women. For instance, two drawings by Judith Warkin in the November 1982 Edinburgh Women’s Liberation newsletter accentuated feminist critiques of biomedicine. One of the drawings depicted four uteri in cages with signs in the background stating, ‘Doctors! Buy your preforming Wombs here!’, ‘Medical students 20% discount’, and ‘men only’. Warkin’s second drawing featured several uteri carrying off a man wearing a white coat and stethoscope with the caption ‘Wombs Fight Back’.^26^ These drawings satirically displayed the feminist charge that medical professionals treated women patients as a disembodied womb, and outlined what many feminists saw as the performative, transactional, and experimental relationship between women and biomedicine. In addition to the written and artistic commentary in the local Women’s Liberation newsletter, some local women’s health activists established a women’s health shop on the city’s Royal Mile where women could go to learn about how to conduct a breast self-examination and get information about their bodies, patient and reproductive rights, and many common health issues.^27^ While less provocative than the Warkin drawings on the surface, the presence of this shop on Edinburgh’s Royal Mile situated women’s health as a key part of Edinburgh’s political, medical, and cultural centre. The location of this shop was within walking distance from many of the University of Edinburgh’s Medical School and the City Council Chambers and centrally located for many locals and tourists, making the shop highly visible to the local medical community, city council employees, as well as the broader public and tourists.

Edinburgh was also home to many national public service organisations and local university programmes that drew in progressive health educators and fuelled women’s health activism in the city. As Amanda Amos, a women’s health organiser and public health researcher, explained in an oral history interview, ‘it was a great place to be in Edinburgh at that time.’^28^ The home of SHEG and the Usher Institutes’ Community Medicine Department at the University of Edinburgh, in particular, made Edinburgh one of the key places where community medicine and health education programmes flourished in 1980s Britain.^29^ Women’s health practitioners and activists increasingly joined these public health spaces and brought women’s health issues to Scottish health agendas. For example, from the late 1970s to the early 1980s M. Maureen Roberts’ clinical trial on mammography and her public health campaign for breast cancer screening and self-examinations brought clinical practice into conversation with the broader feminist praxis of self-examination.^30^ Similarly, women’s health activists like Yvonne Bostock, Una Mclean, and Jane Jones brought feminist health concepts to the growing progressive community health network in Edinburgh, as they earned positions at SHEG, the Usher Institute, and the local community health projects (respectively).

For example, Yvonne Bostock, a women’s health activist and SHEG employee in the late 1970s and early 1980s, had advocated to get women’s health on the organisation’s official public health agenda. Amanda Amos recalled, ‘at that time, it was all about men’s health … Scottish women probably had the worst women’s health, but it was Scottish men. … So, a lot of the stuff SHEG was doing was very much aimed at men and through football.’^31^ Yvonne recalled how she carved out her own space and expertise in women’s health within this male-centric SHEG programming at the turn of the 1980s: ‘a lot of our [SHEG] information maps were all targeted at men, the images were all of men smoking and so on. … I, in a staff meeting raised, all our campaigns are really aimed at men but smoking rates for women have gone up and up and up. Oh right, we should do something for women.’^32^ With the support of SHEG director, David Player, Yvonne reached out to her connections with the local women involved in Action on Smoking Health (ASH), like Amanda Amos and Allison Hillhouse, to create SHEG materials about women and smoking. Using this momentum, Yvonne expanded the women’s health agenda at SHEG to screening and prevention more broadly. She even utilised SHEG’s ‘deal with BBC Scotland’ to run weekly radio segments about women’s health issues, including breast screening services, menopause and family planning, on the Jimmy Mack Show. Yvonne explained that she used the background research and materials produced for the Jimmy Mack show to create information pamphlets on specific women’s health issues, which vastly expanded the SHEG health education materials.^33^

The production of the *Well Woman* book in the early 1980s exemplifies the connections between the women’s health movement and the official public health initiatives in Scotland during the early 1980s. While the book was produced by SHEG and published with no explicit list of individual authors, the acknowledgements stated, ‘The Scottish Health Education Group wishes to acknowledge the large number of people who have contributed their experience, expertise and advice in producing this book.’^34^ In oral history interviews, Yvonne described *Well Woman* as a culmination of her work within and outside of SHEG, and the book included a wide-ranging list of women’s health topics from mental health issues to pregnancy and women’s bodies.^35^ Building on her successes at SHEG with the women and smoking campaigns and the BBC radio segments, Yvonne drew on her connections across the women’s health networks of Scotland to write the *Well Woman* book. She recalled, ‘it just emerged and obviously I’d written all this stuff. … So, it was like, let’s put together a comprehensive thing, so I started work on that and I was using this network of people to get them to draft things as well.’^36^ Yvonne was the only SHEG employee who contributed to *Well Woman*, but she recalled that obstetrician-gynaecologist Dr Nancy Beaton, who ran the local Family Planning Clinic, and Jean Malcom, who ran the Edinburgh Brook Advisory Service, sat on the SHEG advisory board for family planning and advised on several sections of *Well Woman*. Yvonne also reached out to Jill Rakusen and Angela Phillips, the women who adapted the 1978 British edition of *Our Bodies Ourselves* to consult on the production of the *Well Woman* book.^37^ Yvonne’s memories of any women who consulted on, and contributed to, the *Well Woman* book underscore the vibrant women’s health network active in Scotland during the turn of the 1980s.

Despite this flourishing community of women’s health experts in Scotland, feminist health education and activism was still met with resistance and, sometimes, censorship from some medical and political professionals in Scotland. In the early 1980s, controversy about the nature of women’s health education came to a head over the *Well Woman* book. When SHEG initially printed and distributed the *Well Woman* book in early 1983, the Scottish Home and Health Department immediately received complaints from Chief Medical Officers across several regions of Scotland. Yvonne remembers being told that one of the chief medical officers actually burned his shipment of the *Well Woman* books in protest of its content: ‘it went to … one of the Scottish islands and the chief medical officer had them all burnt. … I can remember David calling me into his office and saying, well we’ve just heard that the delivery has been burnt.’^38^ These medical officials took particular offense to one section of the book titled ‘Your intimate body parts,’ an anatomically accurate drawing (see *Image 1*) that identified and distinguished a woman’s vulva, urethra, vagina, and clitoris, alongside written explanations.^39^ After receiving these complaints, the Home and Health Department claimed that the *Well Woman* book was ‘over concerned with sexuality and too strident to be a government document’.^40^ They stopped the production and distribution of the *Well Woman* book and demanded that the book be rewritten.^41^

#### Image 1. Image from Well Woman: A Guide to Women’s Health, 1983, page 29. Source courtesy of Yvonne Bostock

The official political and medical response to the *Well Woman* book demonstrates the stigma surrounding women’s sexuality, health, and bodies that persisted into the 1980s. SWHF organiser Amanda Amos explained the contrast between the ‘quite conservative’ Scottish Office and the supportive ‘small world’ of the women’s health and health promotion communities in Scotland and Edinburgh.^42^ Despite the so-called sexual revolution of the 1960s and 1970s, moral panic about permissive sexuality infused political and public discourse about women’s health and rights. As historians Roger Davidson and Gayle Davis have shown, even by the 1970s and 1980s many Scottish politicians and policy makers saw sex education as an opportunity to emphasise heterosexual normality and morality. They explain that ‘the sexual urge, especially amongst girls, was still depicted as inherently dysfunctional unless controlled and deferred into the socially acceptable, heterosexual contexts of marriage and family formation.’^43^ Kristin Hay’s work has shown that as Scottish legislation and policy became more permissive, at least on the surface, hostile attitudes towards youth and women’s sexuality outside of marriage persisted in some public, political, and medical spaces into the late twentieth century.^44^ And, the *Well Woman* book controversy exemplifies that women’s health activists in Edinburgh, and Scotland more broadly, faced political and medical gatekeeping and knowledge control despite support from their activist and progressive community health communities. The Home and Health Department’s censorship of *Well Woman* and their use of the term ‘strident’ to describe the book sent a message that Yvonne and other feminists at SHEG had overstepped their mark

With the censorship of the *Well Woman* book, women’s health activists in Scotland were in a precarious position. Many had developed their expertise in reproductive, sexual, and women’s health through their grassroots information activism,^45^ and had gained positions in government and government-funded public health services like SHEG or different community health projects. Slighted by the government’s actions, many activists leaned on their grassroots and professional allies to figure out how to pivot their strategies. In their oral history interviews, Amanda Amos and Yvonne Bostock explained that these local and national networks were important for women’s health activists to do their work, especially in the wake of controversy, like the censorship of the *Well Woman* book.^46^

While women’s health activists in Edinburgh faced a dual reality in the 1980s as their strong activist networks clashed with political and medical authorities, local women’s health organisers faced a new challenge: working with the WHO European Region. The WHO staff were impressed with the public health materials that Yvonne Bostock and other women at SHEG had produced, and wanted to join forces with SHEG to co-host a conference on women and health. The WHO’s goal was to bring invited delegates from Europe and North America together to discuss leading women’s health research in Scotland. Yvonne was pulled onto this project as one of SHEG’s women’s health experts. She quickly raised the issue that the WHO’s conference excluded the broad network of women’s health activists in Scotland at an event happening in their own backyard: ‘Because all these things we’ve done together with all these people in Scotland, we can’t do that to them, we cannot, it’s not right.’^47^

After heated discussion between Yvonne, SHEG superiors, and the WHO representatives, it was decided that SHEG would also sponsor a Scottish-women-led health fair, to be held alongside the WHO conference. Even though she did not agree with the WHO organisers’ approach, Yvonne—as the women’s health expert at SHEG—was essential to making the WHO-SHEG conference a reality. Many SHEG employees saw this conference as an important opportunity to gain WHO ‘collaborating centre status’.^48^ To get Yvonne’s cooperation in the WHO-SHEG conference organising, her boss and colleague at SHEG suggested a compromise: SHEG would fund a women’s health fair, separate from the WHO conference, that could be planned and run by women’s health organisers. Yvonne remembered, ‘Iain was saying, “well I think you are being unreasonable, Yvonne. An international conference, just think it’s the WHO, the kudos for Scotland.” And I said, “yes, I know but just think what we can do for the women of Scotland.” … Anyway, I was having none of it and there was this talk of, we’ll have a women’s health fair.’^49^ In its inception, the SWHF was linked to SHEG and the WHO conference. But the internal politics at SHEG surrounding the conference and the fair meant that the SWHF organisers were increasingly left to their own devices to plan and carry out the event.^50^ The SWHF was established within these complex and fraught local, national, and international contexts surrounding the *Well Woman* book and the WHO conference. The robust community of women’s health organisers and the women’s health work at SHEG initially attracted the WHO to Scotland, but this was the same community who felt slighted by the WHO’s exclusive approach to their conference. Tasked with co-organising the WHO conference and spearheading the fair, Yvonne recruited women’s health activists—including Jo Burns, Jane Jones, Faye Jacobsen, Sarah Boyle, Linda Headland, and Jean Malcom—to plan and run the SWHF.^51^ This original planning committee and many others individuals, like Penny Richardson and Amanda Amos, who were recruited throughout the project, designed the fair using feminist and community-led approaches, in an purposeful juxtaposition to the invite-only WHO conference.

### ‘A Means for Change’: The SWHF and Feminist Heath Praxis

In line with broader feminist health activism in the 1970s and 1980s, the SWHF organisers wanted to ensure that the fair would be driven by the concerns of ‘ordinary women’ across Scotland. Community-driven approaches were used by women’s health activists beyond Scotland and, as Dudley-Shotwell has shown, the self-help nature of feminist health spaces attracted ‘women from many walks of life’.^52^ Early meeting minutes of the SWHF planning committee demonstrate that the organisers defined ‘ordinary’ women broadly and hoped to reach Scottish women beyond the urban middle classes of Edinburgh and Glasgow. Meeting minutes from July 1982 quoted Jo Burns, who stated that recruitment materials should be sent to women from ‘isolated crofts and fishing villages to rapidly expanding “oil towns”’.^53^ To ensure diverse perspectives and participation in the fair’s design, the planning committee began mailing letters and recruitment pamphlets to women’s groups across Scotland as early as Summer 1982.^54^ The early recruitment materials for the SWHF highlighted their community design approach. The planning committee created a pamphlet titled, ‘Your Health, Your Health Fair’, to solicit feedback on its design. The pamphlet nodded to the existing women’s health and liberation networks in Scotland as it described the broad goal of the SWHF ‘as an event to launch a nation wide process of continuing contacts and discussion’.^55^ It also underlined that individual Scottish women and organisations would guide the design of the fair. The front cover of the pamphlet stated: ‘FAIR WARNING! Health Fair?! What’s a health Fair?! I don’t know, but there’s going to be one in Edinburgh Next May.’^56^ The pamphlet stated that even the organisers could not say exactly what the fair would look like until they heard from Scottish women about their concerns and ideas, stating that the SWHF design ‘will depend on the activities of thousands of women across Scotland’.^57^

SWHF organisers travelled across Scotland to host information sessions at community centres to follow up the distribution of the ‘Your Health, Your Health Fair’ pamphlet and recruit more presenters and attendees to the fair. Jane Jones remembered they used sketches to attract a broad audience and open discussion about women’s health issues in each community. She stated that she travelled to ‘Aberdeen, and Dundee, and Inverness, I think, and Glasgow … and talked to the women about it, and said, come to the fair. So, we were trying to get them to come through using this sketches thing.’^58^ Throughout 1982 and early 1983, the organisers met with several women’s groups from other urban centres like Glasgow and Aberdeen, smaller cities such as Stirling and Dundee, as well as the towns of Renfrew and Dumfries, where they gathered ideas and priorities for the fair.^59^ With the feedback from women’s health groups across Scotland in mind, the planning committee designed the SWHF to facilitate an exchange of experiences and information, prompt discussion to define key concepts and challenges to women’s health in Scotland and, overall, inspire ‘a means for change’.^60^ This networking across Scotland paid off in the end, as Jane Jones remembered ‘the great bustle’ of the SWHF as ‘loads of women [came] from all over Scotland’—despite the fact that ‘it poured down the whole weekend’.^61^

Even the visual design of the SWHF programme detailed the community-led approach and aims of the fair. The programme cover featured a colourful drawing of two women and two small children (see *Image 2*). The interior of the programme incorporated small drawings of this family alongside information about the event. These drawings, all by Mary McCann, visually followed this family of women and children as they visited various parts of the SWHF: their journey on the bus to Edinburgh, their visit to the information centre for tea, the Kids Play Tent where the two adults leave the children as they begin to participate in fair activities, like yoga sessions and various workshops. While a fun visual choice for a programme, the illustrations of the family’s journey through the programme underscored the core values of the SWHF. The fact that the women pictured travelled from outside of Edinburgh to attend the conference demonstrated the organisers’ commitment to reach a range of women across Scotland; the available childcare made the adult women’s attendance more accessible; the broad variety of activities pictured framed various ways of knowing and learning about health and wellbeing; and the different ages of the women underscore the focus on health issues for women across the lifespan.

#### Image 2. Scottish Women’s Health Fair Programme Cover, 1983. From the Cervical Smear Campaign collection, GD31/9/1/2, Lothian Health Services Archive, University of Edinburgh

In keeping with their goal to create an event that focused on the health priorities of ordinary Scottish women, the planning committee organised and ran a massive health fair. The two-and-a-half-day event was divided into three themed programmes—Head On, Well Women Centre, and Ideas into Action—that ran alongside wellbeing workshops, various entertainment events, and information stalls from over a hundred Scottish groups and organisations. The Head On programme focussed on women and mental health and included several self-help groups, a film screening series, and a dozen workshops on a variety of issues like stress, sleep, sexuality, depression, and addiction.^62^ The Well Women Centre was set up as a service-based feminist health centre and featured workshops about menopause and periods in addition to general conversations about the well women model and what to expect from Well Women Clinics in the UK.^63^ Ideas Into Action combined formal presentations from activist groups across Scotland with informal strategising discussions about how different groups ‘tackled problems such as damp housing, low incomes, racism, unemployment and poor local health services by campaigning, support groups, practical action (food co-ops)’.^64^ Each of these themes was based on the feedback from women they had connected with across Scotland. And the Ideas into Action theme provided a platform for Scottish activists to share experiences and strategies, including workers from a local health centre in the small town of Greenock, feminist unemployment activists from Dundee, and ante-natal health advocates from Oban.^65^

SWHF organisers also leveraged their various personal and career backgrounds to foster collaborations with well-regarded medical and health services in Edinburgh. The women running the fair, like many other grassroots and service-based feminist health groups, came from a variety of class backgrounds and professions. Women like Penny Richardson and Amanda Amos, who were newer to the women’s health organising scene, worked alongside health professionals like Yvonne Bostock and Jo Burns who worked at the Scottish Association for Mental Health. The organisers also collaborated with health professionals at the fair itself. Some medical professionals, even a few in high-profile positions like the Chief Nursing Officer in Edinburgh, sat on panels and ran workshops at the fair.

This mixing of activist and health professional perspectives at the fair made space for nuanced commentary about women’s healthcare in Scotland more broadly. As Hannah Elizabeth has noted in their work, queer health activists often strategically leaned on alliances with healthcare professionals ‘to get lesbian issues on the agenda’.^66^ The fair’s Well Women Centre exemplified the complex nature of bridging professional and lay perspectives. SWHF organisers recognised that the Lothian Health Service had Well Women Clinics which offered important services like cervical screening and breast examinations but critiqued the short 6-minute appointments provided there. They designed their Well Women Centre based on the Well Women Clinic in Manchester that employed a variety of lay- and health-practitioners and spent much more time with clients, co-designing a care plan. The organisers argued that this model ‘allowed women to recognise the validity of their own needs’ and offered a space for women who avoided medical institutions for ‘reasons of class or culture’.^67^ SWHF organisers used the opportunity to articulate their visions for women’s healthcare beyond the limits of their local health services.

### By and For ‘Ordinary Women’: The SWHF as a Showcase of Strength and Support

SWHF organisers aligned with common 1980s feminist health methods and aims, but in 1983 Edinburgh these approaches and the success of the fair had the added benefit of displaying the widespread interest and support for women’s health organising in Scotland. The archival materials identify the main aims of the SWHF as hosting a women’s health event by and for Scottish women, and do not outline explicit strategies to use the event to face off with the Scottish government or the WHO. However, SWHF organisers carefully and consistently articulated their approach to women’s health in response to what they saw as a closed and elite WHO conference. The organisers also evoked the political power of ‘ordinariness’ to position the SWHF as representative of a broad collective of ‘ordinary’ Scottish women beyond the women’s liberation groups in Edinburgh. Understood within the context of the *Well Woman* controversy and the contention with the WHO European Region, the SWHF also functioned as a showcase of the strength of the women’s health movement in Scotland and the broader support for the movement’s aims from ‘ordinary’ Scottish women.

Health fair organisers and some feminist press juxtaposed the SWHF and the WHO conference and accentuated the fair’s Scottish focus and feminist approach. Amanda Amos remembered that many in the women’s movement questioned why the WHO was setting the agenda for women’s health in Scotland. She remembered, ‘I suppose women involved in the women’s movement started saying, well, hang on, this is all for *high heid yins* [Scots for authority figures], what about Scottish women? How is our health going to benefit from this?’^68^ Similarly, a newspaper article by Shiela McNamara advocated for the SWHF as a necessary local and Scottish intervention. She explained that because ‘of the mere four UK [WHO] delegates, only one was a Scot, it was felt that the people most concerned in the matter—women themselves—really ought to have some involvement somewhere.’^69^ The positioning of the SWHF as open to all Scottish women strategically juxtaposed, and implicitly critiqued, the exclusivity of the WHO conference.

SWHF organisers’ consistent messaging that the fair was by and for ‘ordinary’ Scottish women also had a broader political utility beyond the methods of a feminist community-led design. Historian Claire Langhamer has explored the political and cultural importance and utility of ‘ordinary people’ in postwar Britain. Her work, evoking both individuality and a broad collective, demonstrates that ‘ordinariness was a powerful position from which to resist and to challenge authority, to assert rights and to make demands’.^70^ In the case of the SWHF, there was a clear political benefit of framing this fair as by and for ‘ordinary’ women. It served as a showcase of social acceptance and support from women—en masse—across Scotland in the face of censorship and condemnation from the Scottish home office. In doing so, they positioned the SWHF as in touch with the on-the-ground needs, opinions, and feelings of ‘ordinary’ Scottish women beyond that of the politicians and experts of the government and the WHO. The Scottish Home and Health Department may have charged the *Well Woman* book as forceful and inappropriate, but the SWHF demonstrated that the women across Scotland wanted this information and supported women’s health initiatives.

Beyond implied strategies, the sheer size of the SWHF, both in attendance and in the number of events, situates it as a showcase of the strength of and support for the women’s health movement in Scotland. While the extant archival materials from the SWHF do not record specific attendee numbers or demographics, a tenth anniversary reflection on the fair recalls ‘a couple thousand people coming along’ in 1983.^71^ The fair also boasted over one hundred Scottish groups or organisations participating in the information stalls in Princes Street Gardens throughout the weekend. This mass of SWHF participants would have been an unavoidable presence in Edinburgh’s city centre, as they travelled in between the sixteen venues spread across the New Town.^72^ The number of participants demonstrated that ‘ordinary’ Scottish women wanted women’s health information. And even though it was not the intended aims of the health fair organisers, the visibility of the SWHF attendance, collaborations, and programming in Scotland’s capital city functioned as a collective show of support for women’s health initiatives from women across Scotland.

### Conclusion: The Legacy of the SWHF

Oral history interviewees remembered the fair as a vibrant and important point in their own lives and in the Scottish women’s health movement more broadly. Jane Jones recalled ‘we had an enormous fair, actually. And then, the ramifications were incredible.’^73^ Many women who organised and ran the SWHF built careers out of their interests from the fair and brought their feminist approaches into community and public health spaces. Adrienne Sillar, who was a key figure in establishing the SWHF’s Well Women Centre, worked with other fair organisers and local health professionals to make it a permanent, store-front centre for women’s health and wellbeing.^74^ Many of the women who maintained the Well Women Centre produced a popular women’s self-help book called *Peely Wally*.^75^ Jane Jones, who co-ran the Ideas Into Action theme of the fair, continued her work at the Pilton Community Health project, where she established support groups for women on tranquillisers and other women’s health programmes.^76^ Others involved in the Head On stream of the Fair, like Amanda Amos and Penny Richardson, became academics, specialising in women and health and sitting on the Edinburgh City Council’s Health committee respectively. In her interview, Amanda remembered, ‘then what happened in Edinburgh after that was, we said, “well, we’ve got to continue this.”’ She continued to reflect, ‘you only realise afterwards how groundbreaking this was.’^77^ The organisers continued to claim women’s health expertise beyond the fair itself. Their successes in organising it by and for Scottish women ignited continued women’s health organising across community outreach, public health, and health research spaces. The organisers articulated their feminist and community-led approaches to health within the context of the SWHF. In doing so, they claimed expertise in the growing fields of community and public health and brought these approaches into more mainstream and everyday health services in Edinburgh.

The significance of the SWHF as a showcase for the strength of and support for women’s health initiatives in Scotland illustrates the political power of community-led organising in the face of professional and political opposition. The timing of the fair alongside the *Well Woman* censorship and the power negotiations with the WHO European Region inspired the SWHF organisers and shaped their approaches. The organisers’ feminist methods of community-driven goals and programming both aligned with their own broader women’s liberation goals but had the added benefit of garnering wide support from women across Scotland for women’s health initiatives. In doing so, the mass of SWHF attendees demonstrated that Scottish women wanted good information about women’s health and would not be excluded from the national and international conversations about women’s health. Despite government censorship and WHO exclusivity, the ‘ordinary’ women attending the fair created their own massive space to formulate their women’s health politics and visions.

## Footnotes

2 Programme of the Scottish Women’s Health Fair (hereafter SWHF Programme), 1983, G31-9-2, Cervical Smear Campaign Collection (hereafter CSCC), Lothian Health Services Archive (hereafter LHSA), Edinburgh.

3 SHWF Planning Committee meeting minutes, 6 July 1982, GD31-8-1, CSCC, LHSA; Draft Report of SWHF, 6 July 1983, GD31-8-1, CSCC, LHSA.

4 SWHF Programme.

5 Interview with Amanda Amos, 12 March 2024; Interview with Jane Jones, 16 April 2024; Interview with Adrienne Sillar, 26 April 2024.

6 Draft Report of SWHF, 6 July 1983, LHSA; Interview with Yvonne Bostock, 5 April 2024.

7 Nancy Janovicek, *No Place to Go: Local Histories of the Battered Women’s Shelter Movement*, (Vancouver: University of British Columbia Press, 2007); Cathy Moran Hajo, *Birth Control on Main Street: Organizing Clinics in the United States, 1916*−*1939* (Champaign: University of Illinois Press, 2010); Sarah Browne, “‘A Veritable Hotbed of Feminism’: Women’s Liberation in St Andrews, Scotland, c.1968−c.1979”, *Twentieth Century British History* 23, no. 1 (2012): 100−23; Lianne McTavish, “Abortion in New Brunswick”, *Acadiensis* 44, no. 2 (2015): 107−30; Kristin Hay, “‘More than a Defence against Bills’: Feminism and National Identity in the Scottish Abortion Campaign, c. 1975−1990”, *Women’s History Review* 30, no. 4 (2021): 594−612; Karissa Patton and Whitney Wood, “‘Doctors Aren’t Familiar with Your Tissues’: Self-Examination and Feminist Health Activism in 1970s Canada”, *The Canadian Historical Review* 104, no. 2 (2023): 169−97.

8 Janovicek, 3; Hajo.

9 McTavish, 107−30.

10 Hannah J. Elizabeth, “‘The Wild Women of the West (Midlands)’: How LesBeWell Imagined Queer Women’s Health and Its Obstacles in the 1990s through the Pages of *Dykenosis*”, *Contemporary British History* 37, no. 3 (2023): 309−38, 311.

11 Sally Sheldon et al., *The Abortion Act 1967: A Biography of a UK Law* (Cambridge: Cambridge University Press, 2022).

12 Browne, “A Veritable Hotbed of Feminism”, 101−02; see also Hay, “More than a Defence against Bills”.

13 Virginia Berridge, *Health and Society in Britain since 1939* (Cambridge: Cambridge University Press, 1999), 50, 86−8; Virginia Berridge, *Marketing Health: Smoking and the Discourse of Public Health in Britain, 1945*−*2000* (Oxford: Oxford University Press, 2007), 161.

14 Berridge, *Health and Society in Britain*, 56−7, 72−3.

15 Sheldon et al.; Berridge, *Health and Society in Britain*, 52.

16 Berridge, *Health and Society in Britain*, 52−3.

17 *Ibid*., 41.

18 Judith A. Houck, *Looking Through the Speculum: Examining the Women’s Health Movement* (Chicago: University of Chicago Press, 2024), 2.

19 Hannah Dudley-Shotwell, *Revolutionizing Women’s Healthcare: The Feminist Self-Help Movement in America* (New Brunswick: Rutgers University Press, 2020), 7−8; Houck, 2; Lorraine Greaves, *Personal and Political: Stories from the Women’s Health Movement 1960*−*2010* (Toronto: Second Story Press, 2018), 2; Patton and Wood.

20 Sarah Crook, “The Women’s Liberation Movement, Activism and Therapy at the Grassroots, 1968−1985”, *Women’s History Review* 27, no.7 (2018): 1152−68, 1152.

21 Sarah Browne, *The Women’s Liberation Movement in Scotland, c.1968−c.1979* (Manchester: Manchester University Press, 2014), 67.

22 Zoe Strimpel, “Spare Rib, The British Women’s Health Movement and the Empowerment of Misery”, *Social History of Medicine* 35, no. 1 (2021): 217−36; Hay, “More than a Defence against Bills”, 594−612.

23 Hay, “More than a Defence against Bills”, 594−612; Amos interview; Jones interview; Sillar interview.

24 Flyers from the Well Women Centre, Pilton Community Health Project, Craigentinny Health Project, courtesy of Adrienne Sillar.

25 Interview with Gica Loening, 12 April 2024.

26 Judith Warkin Drawings, *Edinburgh Women’s Liberation Newsletter Special Issue on Women and Health*, vol 4, November 1982, 6, 8, Glasgow Women’s Library, Glasgow.

27 Amos interview; Jones interview.

28 Amos interview.

29 Berridge, *Health and Society in Britain*, 88; Una Mclean, *The Usher Institute and the Evolution of Community Medicine in Edinburgh* (Edinburgh: Usher Institute, 1975); Amos interview.

30 Patton and Wood; Houck, 2−4; M. Maureen et al., “Edinburgh Breast Education Campaign on Breast Cancer and Breast Self-Examination: Was It Worth While?” *Journal of Epidemiology and Community Health* 40, no. 4 (1986): 338−43; M.M. Roberts et al., “Edinburgh Trial of Screening for Breast Cancer: Mortality at Seven Years”, *Lancet* 335, no. 8684 (1990): 241−6.

31 Amos interview.

32 Bostock interview.

33 *Ibid*.

34 Scottish Health Education Group (SHEG), *Well Woman: A Guide to Women’s Health* (Edinburgh: Scottish Health Education Group, 1983), 2. File courtesy of Yvonne Bostock.

35 Bostock interview; SHEG, *Well Woman*.

36 Bostock interview.

37 *Ibid*.; Angela Philips and Jill Rakusen, *The New Our Bodies, Ourselves: A Health Book by and for Women* (London: Penguin, 1978).

38 Bostock interview.

39 SHEG, *Well Woman*, 28−9.

40 Margaret Vaughn, “Rebellion over Veto on Health Booklet”, *Sunday Standard*, 20 February 1983.

41 *Ibid*.; Bostock interview.

42 Amos interview.

43 Roger Davidson and Gayle Davis, *The Sexual State: Sexuality and Scottish Governance, 1950*−*80* (Edinburgh: Edinburgh University Press, 2012), 295.

44 See Kristin Hay, “‘The Pill for the Unmarried Girl is Hardly going to Improve her Character’: The Impact of Changing Sexual Behaviours on the Construction of Adulthood in Scotland, c.1968−1980”, in *Adulthood in Britain and the United States from 1350 to Generation Z*, edited by M. Cannon and L. Tisdall (London: University of London Press, forthcoming); Kristin Hay, “‘The Doctor made Clear his Utter Contempt of Me and I can Remember it Still’: Unmarried Women’s Experiences of Accessing the Pill in Scotland c.1968−1980”, *Social History of Medicine* (forthcoming).

45 Cait McKinney, *Information Activism: A Queer History of Lesbian Media Technologies* (Durham: Duke University Press, 2020).

46 Bostock interview; Amos interview.

47 Bostock interview.

48 *Ibid*.

49 *Ibid*.

50 *Ibid*.; Draft Report of SWHF, 6 July 1983, LHSA.

51 SWHF meeting minutes, 25 January 1983, LHSA.

52 Dudley-Shotwell, 7.

53 SWHF meeting minutes, 6 July 1982, LHSA.

54 *Ibid*.

55 *Ibid*.

56 “Your Health, Your Health Fair Pamphlet”, c.1982, G31-9-2, CSCC, LHSA.

57 *Ibid*.

58 Jones interview.

59 SWHF meeting minutes, 6 July 1982, LHSA.

60 SWHF Proposal, 22 June 1982, GD31-9-1, CSCC, LHSA.

61 Jones interview.

62 SWHF meeting minutes, 25 January 1983, LHSA; SWHF Programme.

63 SWHF Programme.

64 *Ibid*.

65 *Ibid*.

66 Elizabeth, 314.

67 Draft Report of SWHF, 6 July 1983, LHSA.

68 Amos interview.

69 Shiela McNamara, article on the SWHF and WHO, c.1983, news clipping from files provided by Yvonne Bostock.

70 Claire Langhamer, “‘Who the Hell are Ordinary People?’ Ordinariness as a Category of Historical Analysis”, *Transactions of the Royal Historical Society* 28 (2018): 175−95.

71 Gica Loening, “The Origins of the Well-Woman Centre: Interview with Fay Jacobson”, in *Women Unlimited Ten Years On: A Women’s Health Project for the Women of Gorgie/Dalry* (Edinburgh: Women Unlimited, 1993): 5−6. File courtesy of Gica Loening.

72 “Highlighting the Healthy Woman”, *The Scotsman*, 25 May 1983.

73 Jones interview.

74 Sillar interview; Loening, *Women Unlimited 10 Years On*.

75 Sillar interview; The Well Women Centre, *Peely Wally: Edinburgh Women’s Health Handbook* (1984), courtesy of Jane Jones.

76 Jones interview.

77 Amos interview.

